# From national strategy to local implementation: a policy analysis of eye health integration and financing in Kenya’s devolved health system

**DOI:** 10.3389/fpubh.2026.1941160

**Published:** 2026-09-07

**Authors:** Joyce Koech, Mary B. Adam, Abimbola Ayeni, Jefitha Karimurio, Babar Qureshi

**Affiliations:** 1 Institute of Healthcare Management, Strathmore University, Nairobi, Kenya; 2 Inclusive Health Initiative, Christian Blind Mission, Nairobi, Kenya; 3 Paediatrics and Community Health Unit, Kijabe Hospital, Kiambu, Kenya; 4 Inclusive Health Initiative, Christian Blind Mission, Bensheim, Germany; 5 Department of Ophthalmology, University of Nairobi, Nairobi, Kenya; 6 Inclusive Health Initiative, Christian Blind Mission, Cambridge, United Kingdom

**Keywords:** eye health, health financing, health policy, Kenya, local implementation, national health strategy, strategic plan

## Abstract

**Background:**

In 2010, Kenya’s Constitution created a devolved governance structure, with county governments assuming responsibility for health service delivery after 2013. Eye health carries a burden, with blindness prevalence at 0.37% and vision impairment affecting 11.0–37.5% across regions. Policy and financing mechanisms shaping service availability remain poorly understood. Kenya has advanced through seven strategic plans, the latest being the National Eye Health Strategic Plan (NEHSP, 2020–2025). While plans provide direction, they have operated without a policy framework, limiting financing, accountability, and sustainability. The Ministry of Health is developing Kenya’s first eye health policy, offering an opportunity to address gaps. We conducted documentary analysis of national and county documents to assess eye health integration and financing in Kenya’s devolved health system.

**Methods:**

Sources included national strategic plans and disease-specific policies, county policies and draft policies, and documents: County Integrated Development Plans (CIDPs), Annual Development Plans (ADPs), and Budget Review and Outlook Papers (BROPs) aligned with the 2023–2027 cycle. Two researchers coded policy documents using indicators from the Walt and Gilson policy triangle framework, reaching consensus on items. Findings apply to seven selected counties.

**Results:**

Nationally, the NEHSP provides direction but does not mandate budget allocations. At county level, one county had a validated policy and strategic plan; two had strategic plans; and four had draft policies. All seven County CIDPs mention eye health, with four establishing budget lines. Six counties included budget estimates in CIDPs or ADPs. However, actual expenditure could not be traced as resources may be embedded within broader allocations. Budget estimates exist, but disbursement or expenditure cannot be confirmed. Moreover, most policies are dated 2025-2026, while fiscal documents were prepared earlier in the 2023–2027 cycle. This temporal mismatch limits linkage between policy adoption and documented allocations.

**Conclusion:**

County-level policy adoption and fiscal visibility remain limited in the seven purposively selected counties. Most evidence is confined to budget estimates rather than documented allocations or expenditure. Kenya’s first national eye health policy is an opportunity to embed financing and accountability provisions. Findings underscore the need for vertical coherence in decentralized systems and suggest an inductive adaptation of the Walt and Gilson policy triangle to capture multilevel governance dynamics.

## 1 Introduction

Over the past decade, eye health has gained increasing prominence within the global health agenda, driven by growing recognition of the burden of visual impairment and the importance of vision in achieving universal health coverage and the Sustainable Development Goals (SDGs) (1, 2). More than 1.1 billion people live with preventable or untreated vision loss, approximately 90% of which can be addressed using cost-effective procedures such as spectacles and cataract surgery (1, 3). Globally, untreated vision loss results in substantial productivity losses (4), highlighting the potential economic returns of investing in eye health. About 90% of those with visual impairment reside in low- and middle-income countries, Kenya included (3).

Kenya’s 2024 Rapid Assessment of Blindness (RAAB) survey found a blindness prevalence of 0.37% among adults aged 50 years and older (presenting visual acuity <3/60 in the better eye) and vision impairment ranging from 11.0 to 37.5% across the survey’s study regions, depending on the definition of impairment used (5). Over 80% of blindness is due to treatable or preventable conditions.

An estimated 7.5 million Kenyans require eye care services (6). The country has only 151 ophthalmologists serving a population of 51 million, with their distribution across counties ranging from none to 17 per million people (7–9).

The World Health Organization (WHO) has called for integrated, people-centered eye care within essential service packages and primary health delivery (3). Similarly, the International Agency for the Prevention of Blindness (IAPB) and the WHO Global Action Plan for Universal Eye Health have sought to translate this vision into national policies and measurable targets (10, 11).

Comprehensive policy frameworks establish service delivery approaches, referral pathways, financing mechanisms, and accountability for service provision (12, 13). Yet eye health policy formulation and implementation remain inconsistent, particularly in low- and middle-income countries (LMICs) (14). In Nigeria, national policies acknowledge eye health, but implementation at the primary health care (PHC) level remains limited, with activities often vertical rather than system-wide (15, 16). Across sub-Saharan Africa, integrated eye care services are hampered by policy fragmentation, weak governance frameworks, and poor coordination between donor and national priorities (17–19).

Critical gaps include weak integration of eye care into primary health systems, inadequate coordination across sectors, insufficient human resources, and the absence of reliable data to inform decision-making (1). Contributing factors include limited evidence on the cost-effectiveness of eye health interventions, fragmented advocacy efforts, competing health priorities, and challenges associated with embedding eye care within broader health and social systems (14, 18, 19).

However, countries that have successfully integrated eye care demonstrate that progress is achievable when evidence, political commitment, and coordinated stakeholder engagement converge (16). Effective national eye health policies require inclusion of eye care within essential health benefit packages, investment in workforce development, and sustainable financing and monitoring systems (20).

Kenya’s 2010 Constitution created one national and 47 autonomous county governments, with policy formulation at national level and implementation at county level. Health is the most significant function devolved to counties (21). Devolution has introduced new administrative and governance dynamics, particularly in the health sector, making it important to study how these changes affect system performance. We analyzed how Kenya’s policy environment influences eye health services by examining existing eye health policies and funding mechanisms. Specifically, we address:

- How is eye health integrated and prioritized across national and county health policy frameworks?
- How do the current financing mechanisms influence resource availability for eye health services?
- How do policy and financing factors relate to the documented implementation arrangements and fiscal visibility of eye health services?

This study makes several novel contributions. First, while Kenya’s devolution and its implications for health systems have been studied broadly, no previous research has systematically examined eye health policy integration and financing across multiple counties in the post-devolution era. Second, the study provides the first empirical analysis of the gap between national eye health strategic planning and county-level fiscal commitments using documentary evidence from both policy and budget documents. Third, methodologically, the study suggests the extension of the Walt and Gilson policy triangle framework to account for multi-level governance in decentralized health systems, with relevance beyond eye health. Fourth, the study is timely: with the Ministry of Health currently developing Kenya’s first eye health policy, this study provides evidence that can inform the design of financing mechanisms, accountability provisions, and vertical coordination structures. The study therefore contributes to health policy analysis literature while offering actionable insights for policy development in Kenya and other decentralized systems.

The NEHSP 2020–2025 identifies several barriers to quality eye care (6). This strategic plan is the seventh in the documented history of eye health in Kenya and has provided important direction for eye health over successive planning periods. The sustained development of strategic plans demonstrates the Ministry of Health’s long-standing commitment to addressing avoidable blindness and vision impairment. However, it is important to recognize that Kenya’s eye health framework has operated through a strategic plan, which is an operational roadmap rather than a policy. The NEHSP creates administrative commitments within the Ministry of Health but does not create obligations for counties. Recognizing this gap, the Ministry of Health is currently developing Kenya’s first ever eye health policy (as of Aug 2026, this process is ongoing), representing a critical opportunity to establish a framework for sustainable eye health delivery. This was a documentary analysis of national and county-level eye health policy, strategic, and fiscal/planning documents. The analysis covered the period from 2010 onward for policy documents, aligning with Kenya’s devolution era and the rollout of universal health coverage. The study aimed to assess the level of eye health integration and financing in Kenya’s devolved health system.

## 2 Methods

### 2.1 Study design

Sources of data included national strategic plans and disease-specific policies, county policies and draft policies, and fiscal documents included County Integrated Development Plans (CIDPs), Annual Development Plans (ADPs), and Budget Review and Outlook Papers (BROPs) aligned with the 2023–2027 cycle. County Integrated Development Plans (CIDPs) are five-year county development frameworks that guide planning and budgeting. Annual Development Plans (ADPs) translate CIDPs into actionable annual budgets, while Budget Review and Outlook Papers (BROPs) review actual expenditure against budgeted amounts. Fiscal and planning documents were aligned with County Integrated Development Plans (CIDPs) active period (2023–2027) to ensure consistency in the analysis of planning, budgeting, and accountability documents. This included CIDPs covering 2023–2027, and the most recent ADPs and BROPs available at the time of the search. ADPs for 2023/2024, 2024/2025, 2025/2026, and National and County BROPs for 2023, 2024, and 2025.

### 2.2 Search strategy

We conducted a documentary search between January and March 2026. Websites of the National Ministry of Health, county governments, the National Treasury, and the office of the Auditor General were systematically searched. Search terms included “eye health,” “eye care,” “ophthalmology,” “ophthalmic services,” “vision,” “blindness,” “cataract,” “trachoma,” “refractive error,” “diabetic retinopathy,” “child eye health,” “strategic plan,” “CIDP,” “county integrated development plan,” “county health strategy,” “Annual Development Plans,” “Budget Review and Outlook papers” “budget,” “county fiscal strategy paper,” “expenditure,” and “audit.” In parallel with the web-based search, a stakeholder-based search strategy was used. Policy stakeholders at the Ministry of Health and counties were contacted telephonically by the first author to obtain any relevant documents not publicly available on websites. All contacts were used exclusively for document acquisition; no formal interviews were conducted and no personal information, opinions, or non-public communications were recorded.

Between April and July 2026, during the write-up phase, documents were re-accessed to verify specific details, quotations, and page numbers. The documentary search was updated in July 2026 to ensure that all ADPs and BROPs available within the CIDP active period were included, allowing for comprehensive analysis across planning, budgeting, and accountability documents over multiple years.

### 2.3 Inclusion criteria

Formally adopted eye health policies and strategic plans were defined as published documents providing a comprehensive framework for eye health delivery. Drafts or unvalidated documents were included as evidence of policy development activity but were not treated as formally adopted policy. Clinical guidelines for eye care were excluded from this analysis.

At the county level, the sample included Bomet County Eye Health Policy and Strategic Plan (both validated); Embu, and Vihiga Eye Health Strategic Plans (validated); and draft policies from Kajiado, Kiambu, Kwale, and Meru.

Fiscal and planning documents included CIDPs, ADPs, and BROP aligned with the CIDP active period (2023–2027). At the national level, BROPs for the same period and the latest National and County Health Budget Analysis (FY 2023/2024) were reviewed. The inclusion was purposive and contingent on the prior identification of a county eye health policy or a draft pre-policy eye health document. The analysis cannot determine whether eye health is more or less visible in the budgets of the remaining counties, as fiscal documents for those counties were not reviewed. Including counties without policies would not have allowed assessment of whether policy intentions translate into fiscal reality, the central question of this analysis.

### 2.4 Data sourcing and screening

#### 2.4.1 Policy documents

The screening and review of documents was conducted by two researchers (JKo and AA), with a final verification and expert review by one researcher (JKa). From an initial pool of 22 policy documents sourced, the final policy sample comprised 12 policy and strategic frameworks including the NEHSP, national disease-specific strategies, and county-level policies/strategic plans. One county draft policy (Meru) was identified and included after the initial analysis during the peer review process A complete account is provided in Table 1. Documents were categorized and reasons for exclusion documented (Supplementary material).

**Table 1:** Policy documents identified and analyzed.

Document category	Document title	Year	Level	Status
National strategic plan	National eye health strategic plan	2020	National	7th in documented history; provides national direction
Disease-specific eye health policies	Kenya national master plan for the elimination of neglected tropical diseases (NTDs)	2022	National	Provides a national framework for the control and elimination of priority NTDs
	The Kenya national breaking transmission strategy for soil-transmitted helminthiasis, schistosomiasis, lymphatic filariasis and trachoma	2019–2023	National	Outlines integrated strategies to interrupt transmission
	The 2nd Kenya national strategic plan for control of neglected tropical diseases	2016–2020	National	Establishes the prevention, control, and monitoring of NTDs
County validated and draft policies and strategic plans	Bomet comprehensive eye health policy	2025	County	Validated policy
	Bomet eye health strategic plan	2025	County	Validated Strategic Plan
	Embu eye health strategic plan	2025	County	Validated; awaiting publication
	Vihiga eye health strategic plan	2025	County	Validated; awaiting publication
	Meru eye health policy	2025	County	Draft awaiting county executive endorsement
	Kajiado eye health policy	2026	County	Draft awaiting sign-off
	Kiambu eye health policy	2025	County	Draft awaiting county assembly approval
	Kwale eye health policy	2025	County	Draft awaiting county executive approval

#### 2.4.2 Fiscal documents

For fiscal documents, the purposive sampling strategy yielded documents from the national level and seven counties. No fiscal documents were reviewed for the remaining counties, as no identifiable policy or pre-policy framework for eye health was found for them.

The final fiscal sample comprised 47 documents:

Seven CIDPs: covering 2023–2027.

Twenty-one ADPs: covering 2023/2024, 2024/2025, 2025/2026.

Fifteen county BROPs: covering 2023, 2024, 2025.

Three national BROPs: covering 2023, 2024, 2025.

One national and county health budget analysis (FY 2023/24) (Table 2).

**Table 2:** Fiscal and accountability documents.

Document type and purpose	Year/Period	Counties/Level
County Integrated Development Plans (CIDPs) Are five-year frameworks that guide development, planning and budgeting in Kenyan Counties	2023–2027	Bomet, Embu, Kajiado, Kiambu, Kwale, Vihiga, Meru
Annual Development Plans (ADPs) Translate county’s CIDPs into actionable annual priorities, projects and actionable budgetary allocations.	2023	Bomet, Embu, Kajiado, Kiambu, Kwale, Vihiga, Meru
	2024	Bomet, Embu, Kajiado, Kiambu, Kwale, Vihiga, Meru
	2025	Bomet, Embu, Kajiado, Kiambu, Kwale, Vihiga, Meru
County Budget Review and Outlook Papers (CBROP) compare counties’ annual spending and revenue against budgeted amounts	2023/2024	Embu, Kajiado, Kiambu, Kwale, Meru
	2024/2025	Bomet, Embu, Kajiado, Kiambu, Kwale, Vihiga, Meru
	2025/2026	Embu, Kajiado, Kiambu
National level Budget Review and Outlook Papers (BROP) an annual fiscal report produced by the National Treasury. It reviews the previous financial year’s actual financial performance, to guide the national budgeting process.	2023/2024 2024/2025 2025/2026	National
National and County Health Budget Analysis (Tracks public resource allocations, evaluates alignment with policy goals like UHC, and measure compliance with financing benchmarks. It promotes fiscal transparency across devolved health units and pinpoints execution	2023/2024	National

### 2.5 Conceptual framework and data operationalization

**Figure 1:**
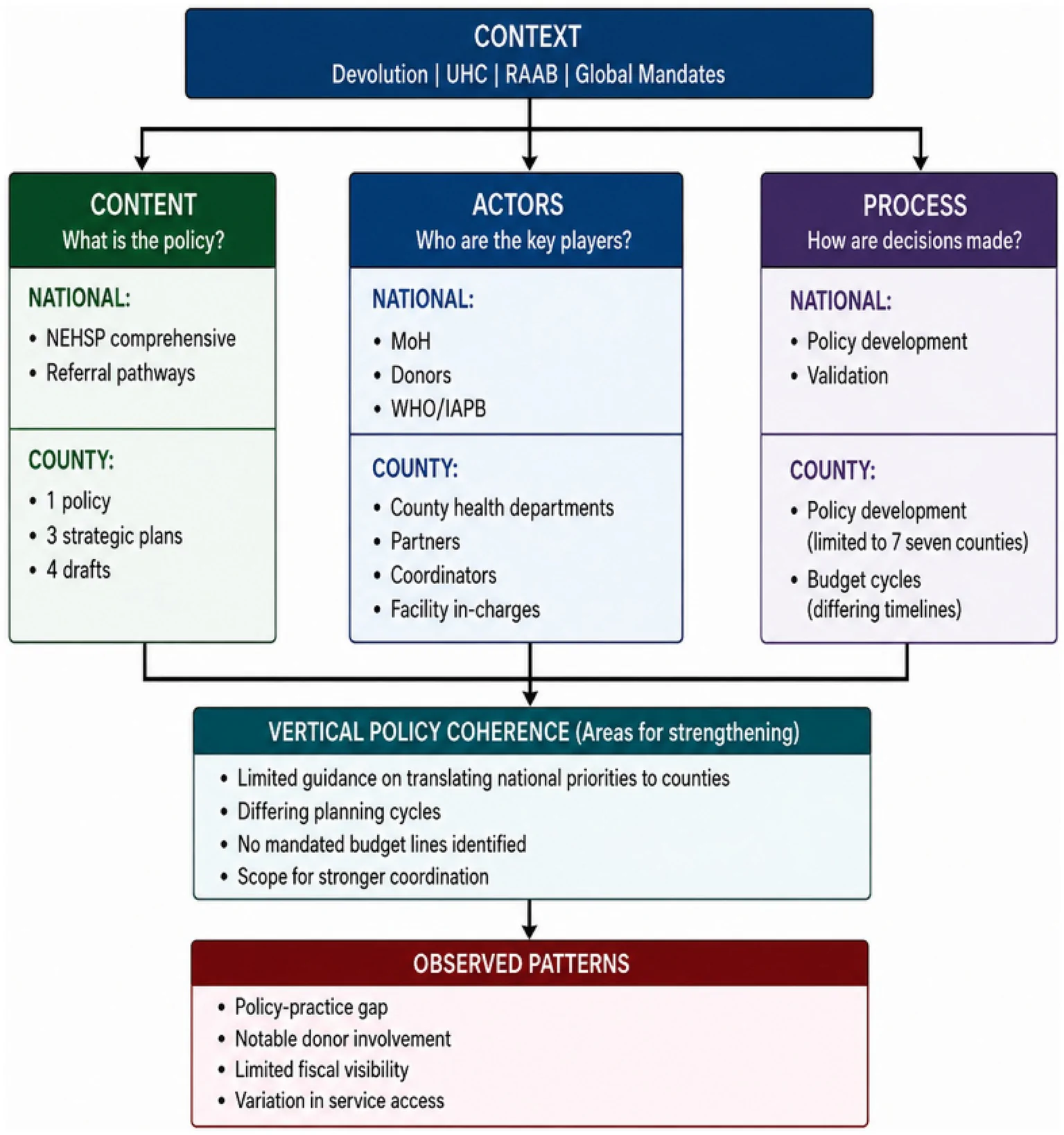
Extended Walt and Gilson policy triangle framework applied to Kenya’s devolved eye health system. Adapted from Walt and Gilson (22), the framework was extended to distinguish between national and county levels of governance. The four policy domains Content, Actors, Process, and Context are shown at each level. Vertical policy coherence (center) emerged inductively during the analysis. It represents the alignment and interaction between national policy formulation and county-level implementation. This distinction captures the difference between policy entitlement (established at national level) and implementation capacity (determined at county level). The framework was operationalized using the 18 indicators described in Supplementary Table S1. Document categories and counts are derived from Table 1 (policy frameworks, *n* = 12) and Table 2 (fiscal documents, *n* = 47). County-level policy chronology status and fiscal visibility are summarized in Supplementary Table S2. Observed patterns (bottom) reflect the findings of the documentary analysis. This framework adaptation requires further application and validation.

To guide the documentary analysis, we adapted the Walt and Gilson (22) policy triangle framework, which conceptualized policy as shaped by four interacting domains: Content, Actors, Process, and Context. Recognizing that Kenya’s devolved governance structure creates distinct policy environments at national and county levels, we extended the framework to distinguish between these two levels. This distinction also reflects the difference between policy entitlement (established at national level) and implementation capacity (determined at county level). The framework was specified *a priori* with national and county domains. However, vertical policy coherence emerged inductively during the analysis as a cross-cutting theme. We subsequently examined it as a mediating dimension between national policy formulation and county implementation (Figure 1). This inductive refinement is proposed as an analytical adaptation requiring further application and validation. We operationalized the framework using document level indicators derived from the policy analysis literature and tailored to the Kenyan eye health context (Supplementary material). The indicators included, Content-presence of explicit eye health goals, referral pathways and M&E indicators; Actors-lead institutions, partners, and evidence of community or civil society engagement, technical working groups; Process-documented stakeholder consultations, validation steps, and coordination mechanisms; Context-references to devolution, broader Kenya health policies, RAAB findings, and alignment with WHO frameworks; Cross-cutting domains- workforce capacity, infrastructure, budget visibility, and Social Health Insurance entitlements; and Risk-donor dependence.

### 2.6 Data extraction and analysis

Two researchers (JKo and AA) independently coded each policy document against these indicators using a pre-specified coding sheet described in Supplementary Table S1. Coding was performed manually using Microsoft Excel, as the indicators were pre-specified and the coding involved identifying concrete content rather than open ended thematic coding. For this reason, no formal intercoder reliability statistic was calculated. Consensus was reached on all items through discussion. Following the coding within each policy triangle domain, cross-cutting patterns were identified and compared across national and county levels to identify vertical coherence or disjuncture. The final themes emerged from this iterative synthesis.

Fiscal and planning documents were independently reviewed by the same two researchers and interrogated for the inclusion of: (1) dedicated budget lines for eye health in planning documents (CIDPs, ADPs); (2) mentions of eye health within broader budget categories; (3) evidence of actual expenditure in accountability documents (BROPs) and (4) any tracking or ring-fencing mechanisms for eye health resources. Consensus was reached on all items. The fiscal review assessed the explicit visibility and traceability of eye health in planning and budget documents; it did not attempt to estimate total eye health expenditure embedded within broader, non-specified budget categories, as this is beyond the scope of this documentary analysis. Budget estimates in planning documents do not constitute evidence for actual disbursement or expenditure. For this analysis, fiscal terminology was defined as follows: budget estimate (projected figure in planning documents), budget line (dedicated entry specifying eye-health resources), allocation (formal designation of resources in approved budgets), expenditure (actual disbursement reported in accountability documents), and fiscal visibility (the presence of eye-health references across any of these categories).

### 2.7 Temporal chronology of documents

An important consideration in interpreting the fiscal findings is the timing of policy development relative to fiscal planning cycles. Supplementary Table S2 provides a chronology for the seven counties. The validated policy and four draft policies are dated 2025. One draft policy is dated 2026. This chronology is essential because fiscal documents may have been prepared before policy documents were developed.

## 3 Results

From an initial pool of policy documents sourced, the final sample included 12 policy and strategic frameworks and 47 fiscal and accountability documents (Tables 1, 2). Others were excluded as they lacked eye health content. The fiscal and accountability documents were obtained for the seven counties that had either a validated county eye health policy or a draft policy or strategic plan.

Policy documents were analyzed using the Walt and Gilson policy triangle. The fiscal and accountability documents were checked for evidence of inclusion of eye health budget lines. Application of the Walt and Gilson policy triangle’s four domains; Content, Actors, Process and Context to the policy documents revealed two cross-cutting patterns. First, analyzing Content across national and county levels exposed a vertical disjuncture between strong national policy and weak or absent county policy adoption. Second, examining Process and Context exposed structural mismatches in planning cycles and devolution.

### 3.1 Conceptual framework application

Figure 1 presents the extended Walt and Gilson policy triangle framework as applied to Kenya’s devolved eye health system. The framework distinguishes between national and county levels of governance and illustrates the four policy domains - Content, Actors, Process, and Context at each level. The vertical axis represents policy coherence between the two levels, highlighting areas where coordination mechanisms were limited or absent from the documents reviewed.

The framework was operationalized using the indicators described in Supplementary Table S1, which guided the coding of all policy and fiscal documents. The Status and Provenance sheet (Supplementary material) provides an overview of policy status and fiscal visibility across these seven counties.

As shown in Figure 1, the analysis revealed a contrast between comprehensive national policy content and limited county-level policy adoption. At the national level, the NEHSP (2020–2025) articulates clear objectives, referral pathways, and monitoring frameworks. At the county level, one county (Bomet) had both a validated policy and a validated strategic plan; two counties (Embu and Vihiga) had validated strategic plans; four counties had drafts, and 40 counties had no identifiable policy framework. Across all 47 fiscal documents reviewed, a nuanced pattern emerged. All seven CIDPs contain explicit mention of eye health, with some counties including budget estimates. However, no accountability documents contained any mention of eye health in the seven purposively selected counties.

The vertical coherence section of the framework captures several structural considerations identified in the analysis: limited guidance on translating national priorities to counties, differing planning cycles between the NEHSP (2020–2025) and CIDP (2023–2027), and the absence of mandated budget lines or intergovernmental coordination mechanisms in the documents reviewed. These factors appear to be associated with the observed patterns of donor dependence and variation in service access across counties, though this study did not directly assess expenditure execution, facility readiness, or patient access.

### 3.2 Policy and strategic plan findings

#### 3.2.1 Comprehensive national frameworks, nascent county-level adoption

The NEHSP (2020–2025) provides comprehensive content, including priority interventions, referral pathways, and monitoring indicators. This strategic plan is the seventh in the documented history of eye health in Kenya (6). The successive strategic plans have served an important function in guiding investments, coordinating partners, and setting direction for the sector. However, it is important to note that Kenya has been implementing eye health activities through successive strategic plans without an underlying statutory policy framework. Under Kenya’s public finance legal framework, funding follows mandates, and mandates derive from statutory policy (23). Strategic plans, even when comprehensive, do not create the legal mandates necessary to trigger budget allocations. The NEHSP creates administrative commitments within the Ministry of Health but does not create legally enforceable obligations for counties. In the absence of an explicit statutory eye-health policy, counties may lack a clear mandate to prioritize eye-health resources. While some counties have included estimates or budget lines in planning documents, the absence of statutory provisions weakens the consistency, accountability, and traceability of such allocations. The absence of a policy appears to be associated with the limited county-level adoption and fiscal invisibility observed in this analysis. This is consistent with documentary evidence; for example, the NEHSP states that “Eye health receives government support at the national level and National Strategy for Eye Health are annexed to the national health sector strategic plan. The Ophthalmic Services Unit at the Ministry of Health develops annual operating plans and budgets based on the National Strategy for Eye Health. These identify the activities covered by health ministry funding and the activities for which external support is required” (6, p. 14). This indicates that the plan itself recognizes the distinction between government-funded activities and those dependent on external support.

At county level, one county (Bomet) had both a validated county eye health policy and a validated strategic plan. Two counties (Embu, Vihiga) had validated strategic plans. Four counties (Kajiado, Kiambu, Meru, Kwale) had draft policies. No publicly available or stakeholder identified policy was found for the remaining 40 counties. While the NEHSP set a target of 2024 county adoption, this goal had not been achieved as of 2026. National policy acknowledges devolution but provides no guidance on how counties should translate national priorities into local policy. Disease specific policies for trachoma (within NTD frameworks) address mass drug administration but do not cover surgical services or broader eye health system integration.

The absence of validated county-level policies means there is no documented framework to guide how eye health services should be delivered, staffed, or funded at the local level, potentially weakening planning, accountability, and sustained eye health service availability in counties without an identifiable policy framework. Notably, even counties with validated strategic plans lack the legal mandate to enforce budget allocations, as strategic plans do not mandate budgets. Bomet’s validated policy is a notable exception yet even though this has not translated into fiscal accountability, as discussed below.

#### 3.2.2 County policy and strategic plan content and proposed financing mechanisms

The validated and draft county policies and strategic plans articulate similar content: they outline service delivery approaches, referral pathways, human resource requirements, and governance structures. For example, the Bomet policy commits to establishing dedicated financing mechanisms, stating as a key strategy to “Have a specific budget line allocated to eye health care services in the county and FIF” (Bomet County Comprehensive Eye Health Care Policy, 2025, p. 13) (24). The policy acknowledges the current gap, noting in its SWOT analysis the “Absence of a defined budget line for eye care services” (24, p. 10). Importantly, county-level documents for all the seven counties propose specific financing mechanisms, including:

1. Dedicated budget lines for eye health within county health budgets.
2. Ring-fencing of Facility Improvement Fund (FIF) resources for eye health services.
3. Mobilization of the Social Health Insurance Fund (SHIF) for eye health services.

However, as detailed in the fiscal analysis below, these proposed mechanisms were not reflected in the corresponding fiscal documents. In pre-policy documents, this may reflect the absence of a policy mandate at the time of preparation. In post-policy documents, the absence of allocations may suggest that policy adoption has not yet translated into fiscal commitments.

#### 3.2.3 Policy adoption and fiscal visibility: financing mechanisms in county budgets

All seven CIDPs contain explicit mention of eye health, demonstrating that counties are already recognizing eye health in their planning documents, regardless of whether they have a validated policy. The fiscal commitments associated with these mentions fall into three categories:

Budget estimates included: Four counties (Embu, Kiambu, Meru and Kwale) have included identifiable budget estimates for eye health in their CIDPs.

Budget estimates embedded: Three counties (Bomet, Kajiado, and Vihiga) have eye health budget estimates embedded within broader programs.

Annual budget estimates across multiple years: Across the 21 ADPs reviewed, five counties (Bomet, Embu, Kajiado, Meru, and Kiambu) demonstrate annual budget estimates or documented achievements.

Six of the seven counties have documented budget estimates for eye health in their CIDPs or ADPs, suggesting that fiscal recognition of eye health is emerging independently of formal policy adoption. This includes Bomet, which despite having a validated policy, has not translated this into a dedicated, separately identifiable budget line.

Critically, actual eye-health expenditure could not be separately verified or traced in the accountability documents reviewed, as resources may be embedded within broader allocations. Budget estimates exist, but disbursement or expenditure cannot be confirmed. This observation was consistent regardless of whether counties have validated or draft policies. BROPs are accountability documents that review actual expenditure against budget. The absence of eye health in these documents means that even where counties have included budget estimates in CIDPs and ADPs, there is no evidence that these funds were actually disbursed or spent as they are not separately verifiable.

#### 3.2.4 Structural timing mismatch between national and county planning cycles

The NEHSP (2020–2025) called for integration of eye health into CIDPs. However, the current CIDP cycle (2023–2027) was finalized before most counties had even drafted their eye health policies. This structural timing mismatch means that even counties with draft policies could not integrate eye health into the current CIDP cycle, as the planning window had closed. Whether these recently developed policies will be integrated into the next CIDP cycle (2028–2032) with corresponding fiscal allocations remains an open question for future observation.

## 4 Discussion

### 4.1 Summary of main findings

This study analyzed how eye health is integrated and prioritized across Kenya’s national and county policy frameworks, examined funding mechanisms, and assessed how policy and financing shape service availability. Three main findings emerge. First, while Kenya has sustained eye health planning through seven successive strategic plans reflecting long-standing government commitment, at county level, one county (Bomet) had both a validated policy and a validated strategic plan; two counties (Embu and Vihiga) had validated strategic plans; and four counties (Kajiado, Kiambu, Kwale, Meru) had draft policies. Second, budget estimates for eye health are emerging at the county level even without validated policies. Third, a structural timing mismatch between the NEHSP cycle (2020–2025) and CIDP cycle (2023–2027), has constrained integration of eye health into county planning. In addition, the absence of eye health in all accountability documents reviewed regardless of policy status makes it difficult to confirm whether these budget estimates were actually disbursed or spent, representing an accountability gap. Together, these findings reveal a persistent translation gap between national strategic ambition and county-level implementation, a gap that the ongoing development of Kenya’s first eye health policy presents an opportunity to address.

Returning to our three research questions: The analysis demonstrates that eye health is integrated into national frameworks but not effectively prioritized at county level. Financing mechanisms exist in planning documents but lack accountability, with no evidence of actual expenditure. Policy and financing factors relate to documented implementation arrangements in specific ways. National service-delivery architecture is clearly articulated. However, county-level operational frameworks remain nascent, and fiscal visibility is limited. These patterns suggest a potential translation gap between national policy intentions and county-level implementation and fiscal commitments.

### 4.2 National policy vs. local implementation

Kenya’s devolved governance structure assigns health service delivery to counties, while the national government retains policy and regulatory functions. Our analysis reveals that while national strategic guidance is comprehensive, county-level policy adoption is limited. This pattern is not unique to Kenya, similar challenges have been documented in Nigeria, where national policies acknowledge eye health but implementation at primary care level remains limited (15), and in South Africa’s KwaZulu-Natal, where eye health is subsumed under broader NCD policies (25).

What This Means for Implementation: Without county-level policies, there is no documented framework to guide how eye health services should be delivered, staffed, or funded locally. This potentially weakens planning, accountability, and service availability. The NEHSP’s target of county policy adoption by 2024 was not achieved, and the national plan provides no guidance on how counties should translate priorities into local frameworks. Even where counties have developed strategic plans, these remain operational documents rather than legally enforceable frameworks.

### 4.3 Financing and accountability

The Fiscal analysis reveals that budget estimates for eye health are emerging at the county level even without validated policies. All seven counties have included eye health in their CIDPs, with four establishing dedicated budget lines and three embedding budgets within broader programs. Six of the seven counties have documented annual budget estimates or achievements in their ADPs. However, it is difficult to confirm whether these funds were actually disbursed or spent.

This limited visibility carries three consequences. First, it undermines accountability. The Health Sector Budget Transparency Review (2023) (26) documented that without distinct budget lines, civil society and oversight bodies cannot monitor whether allocated funds reach intended services. Second, it may reinforce donor dependence. Bomet’s ADP 2025/2026 notes “Ophthalmic services affected by dwindling partner support” (p. 138). The absence of government accountability for eye health expenditure is associated with reliance on external funding. Third, it obscures equity. Without visibility into county-level allocations and expenditure, it is difficult to determine whether resources are distributed according to need, particularly given the wide variation in blindness prevalence across counties (ranging from 11.0 to 37.5% vision impairment (5)).

The implication is that budget estimates in planning documents are insufficient without corresponding accountability mechanisms. The forthcoming national eye health policy should not only mandate budget estimates but should also require reporting on eye health expenditure in annual accountability documents.

### 4.4 Broader implications for health systems

Kenya’s experience offers lessons for other decentralized health systems. The seven successive eye health strategic plans at the National level have provided direction and coordination, but strategic plans alone do not mandate budgets or ensure accountability. This reliance on plans without policy may be associated with donor dependence, as governments struggle to mobilize domestic financing. The ongoing policy development process is therefore pivotal, with implications for financing sustainability, service availability, and rights-based access.

The observed patterns likely reflect a combination of factors. At the county level, competition from other health priorities, particularly infectious diseases and maternal health may divert attention from non-communicable conditions like eye health (27). Fiscal constraints further limit resource allocation, with counties often prioritizing visible infrastructure over preventive services. Workforce shortages compound these challenges, as the uneven distribution of ophthalmologists (8) means that even where budgets exist, service delivery capacity may be lacking.

Additionally, the limited advocacy for eye health relative to other health issues, combined with fragmented planning and budgeting processes and insufficient costing evidence (28), may reduce the fiscal visibility of eye health in county budgets. These factors may operate alongside or in some cases instead of the absence of a statutory national policy.

Health system strengthening requires not only operational planning but also legal frameworks that codify entitlements and mandate resource allocation. The WHO and other global health actors have emphasized the importance of statutory frameworks for durable health system strengthening (29, 30). Kenya’s experience illustrates that sustained strategic planning, while valuable, is insufficient without policy foundations.

Under the Social Health Insurance Act (2023), the Social Health Authority (SHA) administers the health benefits package, while the Social Health Insurance Fund (SHIF) pools contributions to finance covered services. Eye health is embedded within this benefit package. The package defines tariffs for consultation, diagnostic tests, and eyeglasses for children under 18, capped at KES 1,000 per household, and includes ophthalmic surgeries within the broader surgical services package (31). This represents a legal entitlement for citizens, yet actual service availability is uneven due to implementation capacity constraints. Counties without ophthalmologists or equipped theaters cannot operationalize surgical entitlements, and the absence of visible county budget lines may constrain planning, procurement and accountability of optical benefits. The uneven distribution of ophthalmologists (0 to 17 per million people) (6–8) further compounds this gap. A county with no ophthalmologists cannot operationalize a referral pathway requiring cataract surgery, regardless of national policy intent. Thus, even with SHI tariffs, Kenya’s intended eye care service delivery architecture remains aspirational in most counties.

Kenya’s national eye health frameworks articulate a clear service delivery architecture. The NEHSP describes a tiered system with primary health care for screening and basic services, county hospitals for cataract surgery and secondary care, and national referral hospitals for specialized services. Broader health policies such as The UHC Policy (2020–2030), The Kenya Health Policy (2014–2030) and Kenya Community Health Strategy (2020–2025) do not explicitly mention eye health but implicitly cover it under disability and non-communicable diseases (32–34).

### 4.5 Methodological and theoretical contribution

This study suggests the extension of the Walt and Gilson policy triangle to account for multi-level governance in decentralized health systems. The original framework conceptualizes context, content, actors, and process at a single level. We specified national and county domains *a priori* to reflect Kenya’s devolved structure. During the analysis, vertical policy coherence emerged inductively as a cross-cutting theme. We therefore incorporated this theme as a mediating dimension between national and county levels (Figure 1). This represents an inductive refinement of the framework based on the documentary evidence reviewed. Policy analysis in decentralized health systems may benefit from explicit attention to vertical policy coherence. Coherence requires alignment of content, context, actors, and processes across levels of government. Where vertical coherence is absent, national policy ambitions may not be fully realized. This adaptation is proposed as an analytical framework requiring further application and validation.

### 4.6 Comparison with other LMICs

The pattern we observe in Kenya reflects patterns observed in other LMICs. In Nigeria, the National Health Policy (2016) advocates integrating eye care services into existing national health programs, including PHC, yet implementation remains limited and vertical rather than system-wide (15). In South Africa’s KwaZulu Natal, eye health was subsumed under broader NCD policies, with cataract surgery rates the only indicator used to determine financial allocation (25). Tanzania successfully integrated eye health into Integrated Management of Newborn and Childhood Illness (IMNCI), achieved through collaborative research and collective action (35). Ethiopia faces limited funding, human resource shortages, and weak referral systems, leading authors to conclude that ownership, financing, and partnerships need to be strengthened (36).

Kenya’s distinction lies in devolution, the constitutional separation of policy formulation (national) from implementation (county). Unlike other countries where gaps reflect capacity or priorities, Kenya’s devolution makes the structural dimensions especially visible. Without mechanisms for vertical policy coherence, aligned planning cycles, fiscal visibility, and legislative validation, national eye health frameworks risk remaining aspirational. However, direct cross-country comparison of causal factors was beyond the scope of this study.

## 5 Conclusion

Despite comprehensive national eye health strategies, county-level policy adoption and fiscal visibility remain limited, contributing to donor dependence and limiting accountability. Devolution was intended to bring services closer to citizens, but in the absence of strong vertical coordination mechanisms and a clear national policy mandate, national eye health priorities are not automatically translated into county planning, budgeting and implementation. The findings apply to the seven purposively selected counties. No conclusions were drawn about actual expenditure, service availability, or patient access.

The extended Walt and Gilson framework applied in this analysis (Figure 1) highlights the importance of vertical policy coherence in decentralized systems and suggests that policy analysis frameworks must account for multi-level governance dynamics to fully capture the factors shaping policy outcomes. The framework extension is proposed as an adaptation requiring further application and validation. Strengthening county-level policy development, embedding eye health in budgets, and ensuring vertical coherence are essential for sustainable financing and implementation.

## 6 Limitations

First, the fiscal analysis was restricted to seven purposively selected counties that had either a validated or draft eye health policy. This means that the findings cannot be generalized to all Kenyan counties. The absence of a comparator sample of counties without eye-health policies limits the ability to determine whether fiscal recognition can emerge independently of formal policy adoption. Future research should address this gap by including counties without identified eye-health policies to assess whether fiscal visibility arises in the absence of policy activity. Second, we analyzed only publicly available documents or drafts accessible from policy stakeholders. It is possible that other counties have developed eye health policies or implementation plans that were not accessible. However, we argue that policies intended to guide public service delivery should be publicly accessible. Third, we did not conduct interviews with policy actors. The findings reflect the formal documented policy environment and the framework operationalization described in Supplementary Table S1; they do not capture informal practices or political dynamics that may influence policy implementation. Fourth, the study did not assess actual service delivery, county level expenditure execution, facility readiness, patient access, or informal eye health activities. Fifth, our fiscal analysis was limited to explicitly identifying eye health budget lines; we could not capture eye health resources embedded within broader allocations. The findings should therefore be interpreted as evidence of policy and fiscal visibility rather than as a complete assessment of service availability. Sixth, there is a temporal mismatch between policy dates and fiscal documents: most draft and validated policies are dated 2025-2026, whereas fiscal documents reviewed were prepared earlier in the 2023–2027 cycle. This limits the ability to directly link policy adoption to fiscal commitments. Future research should complement this analysis with qualitative inquiry into the barriers and enablers of county-level policy adoption.

## 7 Implications for policy and practice

*National policy mandate*: The statutory eye health policy should consolidate lessons from past plans, establish financing and accountability mandates, and require reporting of actual expenditure. Program-based budgeting with identifiable outputs should ensure traceability.

*Next, CIDP cycle measures*: Counties should adopt eye health policies, supported by gap-assessment tools. Fiscal visibility should be achieved through mechanisms such as distinct budget lines, tagged expenditure, or subprogram codes.

*County implementation requirements*: Coordination mechanisms should link national policy to county action. County health teams should embed eye health in annual workplans and budgets.

*Immediate measures*: Draft County policies should be validated and adopted. The Ministry of Health should guide counties in translating national priorities locally.

*Longer-term monitoring indicators*: County budget allocations and expenditure should be regularly monitored. Donor support should align with county policy development, strengthening capacity rather than substituting government commitment.

## Data Availability

The original contributions presented in the study are included in the article/Supplementary material, further inquiries can be directed to the corresponding author.
