# Supplementary material for: From national strategy to local implementation: a policy analysis of eye health integration and financing in Kenya’s devolved health system
Source: Front Public Health. 2026 Sep 7;14:1941160. doi: 10.3389/fpubh.2026.1941160 (PMC13590745; doi:10.3389/fpubh.2026.1941160)
Supplement: Supplementary file 1 [file Data_Sheet_1.PDF]

Code	Definition	Document indicators	Data source examples	Example extract
Content: Objectives	Presence of explicit eye-health goals or targets	Statement of objectives; numeric targets	NEHSP 2020–2025; county eye policy drafts	Reduce avoidable blindness by X% by 2025.
Content: Service Model	Defined tiered service delivery and referral pathways	Description of PHC → county → referral roles	NEHSP 2020–2025; Bomet County policy	Primary care for screening; county hospitals for cataract surgery.
Content: M&E Indicators	Specified monitoring and evaluation metrics for eye health	Listed indicators; reporting frequency	NEHSP M&E annex; CIDP indicator lists	Cataract surgical rate per 100,000; spectacles dispensed.
Content: Financing Commitments	Explicit budgetary or financing commitments for eye health	Dedicated budget lines; costing; tariff references	SHI Tariffs 2023; county BROP excerpts	KES 935 for consultation and dispensing of eyeglasses.
Actors: Lead Institutions	Named institutional leads and technical units	Named ministry units; professional bodies	MOH Ophthalmic Services Unit; Kenya Ophthalmologi	Ophthalmic Services Unit will coordinate implementation.
Actors: Partners	Identified implementing partners and donors	NGOs; donors; faith-based orgs named	NEHSP partner list; NTD plans	Sightsavers and Fred Hollows Foundation to support training.
Actors: Community Engagement	Evidence of service-user or civil society involvement	References to consultations; community roles	County policy drafts; NEHSP consultation notes	Community health volunteers to conduct vision screening.
Process: Consultation	Documented stakeholder consultation and validation steps	Workshop reports; validation dates	NEHSP development notes; county validation minute:	Draft validated at county stakeholder workshop, June 2024.
Process: Planning Alignment	Alignment with county planning cycles and CIDPs	CIDP cross-references; timeline alignment	CIDP 2023–2027; NEHSP timeline	Integrate eye health into CIDP 2023–2027.
Process: Coordination Mechanisms	Formal intergovernmental coordination arrangements	Joint committees; TWGs; MOUs	NEHSP; county policy MOUs	Intergovernmental technical working group to be established.
Context: Devolution	Explicit reference to devolved governance and implications	Statements on national vs county roles	NEHSP; Constitution references	Policy formulation national; implementation county.
Context: Epidemiology	Use of RAAB or other evidence to justify priorities	Cited surveys; prevalence figures	RAAB 2024; NEHSP references	RAAB 2024: 0.37% blindness prevalence.
Context: Global Alignment	Alignment with WHO/IAPB frameworks and targets	Citations of WHO reports or Global Action Plan	NEHSP references to World Report on Vision	Aligned with WHO Global Action Plan for Eye Health.
Capacity: Workforce	Statements on workforce numbers, distribution, training	Targets for ophthalmologists; optometrists; nurses	National HR plan; county workforce sections	Increase paediatric ophthalmologists to X by 2025.
Capacity: Infrastructure	Equipment and facility readiness for eye services	Lists of required equipment; facility tiers	Facility checklists in county policies; NEHSP	District hospitals to have slit lamp and operating theatre.
Financing: Budget Visibility	Evidence of explicit budget lines or tracking mechanisms	Line items in BROP, ADP, CIDP; FIF ring-fence	County BROP excerpts; CIDP tables	No dedicated budget line for eye health identified.
Financing: SHI Entitlements	Presence of SHI tariffs and access rules for eye services	Tariff amounts; age limits; preauthorization rules	SHI Tariffs 2023 document	Eyeglasses limited to beneficiaries below 18 years; KES 1,000 cap.
Risk: Donor Dependence	Reliance on external funding for service delivery	Statements of donor funding; program dependence	NEHSP partner funding notes; NTD plans	Trachoma programs largely donor funded.
