# Supplementary material for: From national strategy to local implementation: a policy analysis of eye health integration and financing in Kenya’s devolved health system
Source: Front Public Health. 2026 Sep 7;14:1941160. doi: 10.3389/fpubh.2026.1941160 (PMC13590745; doi:10.3389/fpubh.2026.1941160)
Supplement: Supplementary file 2 [file Data_Sheet_2.PDF]

| County | Policy drafting date | Validation date/ Adoption date | CIDP Period | ADP Financial Year | BROP financial Year | Fiscal documents Preceded/ followed policy adoption |
|---|---|---|---|---|---|---|
| Bomet | 2024 (Policy Plan)<br><br>2025 (Strategic Plan) | Validated & adopted 2025 | 2023–<br><br>2027 | 2023/2024,<br><br>2024/2025,<br>2025/2026 | 2023,<br><br>2024,<br>2025 | Preceded |
| Vihiga | 2025 | Validated 2026 not adopted | 2023–2027 | 2023/2024, 2024/2025, 2025/2026 | 2023, 2024, 2025 | Preceded |
| Embu | 2025 | Validated 2026 not adopted | 2023–<br><br>2027 | 2023/2024,<br><br>2024/2025,<br>2025/2026 | 2023,<br><br>2024,<br>2025 | Preceded |
| Kajiado | 2025 | Not yet | 2023–<br>2027 | 2023/2024,<br>2024/2025,<br>2025/2026 | 2023,<br>2024,<br>2025 | Preceded |
| Kiambu | 2025 | Not yet | 2023–<br>2027 | 2023/2024,<br>2024/2025,<br>2025/2026 | 2023,<br>2024,<br>2025 | Preceded |
| Kwale | 2025 | Not yet | 2023–<br>2027 | 2023/2024,<br>2024/2025,<br>2025/2026 | 2023,<br>2024,<br>2025 | Preceded |
| Meru | 2026 | Not yet | | 2023/2024,<br>2024/2025,<br><br>2025/2026 | 2023,<br>2024,<br><br>2025 | Preceded |
